# Electroresponsive Polyelectrolyte Brushes Studied by Self-Consistent Field Theory

**DOI:** 10.3390/polym12040898

**Published:** 2020-04-13

**Authors:** Boris M. Okrugin, Ralf P. Richter, Frans A. M. Leermakers, Igor M. Neelov, Ekaterina B. Zhulina, Oleg V. Borisov

**Affiliations:** 1 CIC biomaGUNE, Biosurface Lab, Paseo Miramon 182, 20014 San Sebastian, Spain; borisokrugin@gmail.com (B.M.O.); R.Richter@leeds.ac.uk (R.P.R.); 2 Institut des Sciences Analytiques et de Physico-Chimie pour l’Environnement et les Matériaux, UMR 5254 CNRS UPPA, 64053 Pau, France; 3 School of Biomedical Sciences, Faculty of Biological Sciences, School of Physics and Astronomy, Faculty of Engineering and Physical Sciences, Astbury Centre for Structural Molecular Biology, and Bragg Center for Materials Research, University of Leeds, Leeds LS2 9JT, UK; 4 Physical Chemistry and Soft Matter, Wageningen University, 6703 NB Wageningen, The Netherlands; frans.leermakers@wur.nl; 5 St. Petersburg National University of Informational Technologies, Mechanics and Optics, 197101 St.Petersburg, Russia; igorneelov@yahoo.co.uk (I.M.N.); kzhulina@hotmail.com (E.B.Z.); 6 Institute of Macromolecular Compounds of the Russian Academy of Sciences, 199004 St. Petersburg, Russia

**Keywords:** smart interfaces, polyelectrolyte brushes, self-consistent field theory

## Abstract

End-grafting of polyelectrolyte chains to conducting substrates offers an opportunity to fabricate electro-responsive surfaces capable of changing their physical/chemical properties (adhesion, wettability) in response to applied electrical voltage. We use a self-consistent field numerical approach to compare the equilibrium properties of tethered strong and weak (pH-sensitive) polyelectrolytes to applied electrical field in both salt-free and salt-containing solutions. We demonstrate that both strong and weak polyelectrolyte brushes exhibit segregation of polyions in two populations if the surface is oppositely charged with respect to the brush. This segregation gives rise to complex patterns in the dependence of the brush thickness on salt concentration. We demonstrate that adjustable ionization of weak polyelectrolytes weakens their conformational response in terms of the dependence of brush thickness on the amplitude of the applied voltage.

## 1. Introduction

Layers of ionically charged macromolecules (polyelectrolytes) tethered by terminal segments onto solid-liquid interfaces (so-called polyelectrolyte brushes) find multiple technological applications because they allow for efficient controlling of adhesive (wettability), tribological and (bio)interactive properties of surfaces operating in aqueous environment [1,2,3,4].

Because of the presence of ionically charged monomer units, polyelectrolyte brushes are capable to undergo drastic conformational changes in response to variation in environmental conditions, such as solution pH or ionic strength. Therefore, modification of solid surfaces exposed to liquid (aqueous) environment by layers of tethered polyelectrolytes is considered as a promising way for the fabrication of smart, stimuli-responsive materials.

During the past decades, a robust “grafting from” approach based on surface-initiated radical polymerization has been extensively exploited to create well-defined dense polyelectrolyte brushes [5]. The results of experimental studies on the structural and adhesive properties of polyelectrolyte brushes and their response to external chemical stimuli were rationalized on the basis of the existing theories [6,7,8,9,10,11,12,13,14,15,16,17,18,19].

While for strong polyelectrolytes it is only the ionic strength of the solution that mediates screening of electrostatic interactions, and governs contraction of the brush, for weak (or pH-sensitive) polyelectrolytes the pH becomes an important control parameter because it determines the fraction of charged monomer units and thus the strength of intermolecular ionic interactions in the brush.

Many applications require instantaneous switching of the interface properties in response to a proper external trigger. However, the conformational response of the nanoscale thick polyelectrolyte brushes to changes in the chemical composition of the surrounding macroscopic solution is unavoidably retarded by the diffusion process and may take minutes. In a search for instantaneous triggers, light is one of the most promising candidates. For instance, it was demonstrated, that UV radiation can cause local acidification of the aqueous solution in the immediate vicinity of titanium dioxide surfaces [20]. If the brush of weak polyacids (or polybases) is attached to such surface, UV irradiation may cause rapid collapse (or swelling) of the brush. Another approach to light-responsive polyelectrolyte surfaces is to the so called photoacids [21,22] in which the ionization constant and thereby the fraction of charged monomer units can be modulated by illumination.

Electrical voltage applied to a conducting (or semi-conducting) substrate decorated with a polyelectrolyte brushes may serve as an alternative external trigger for manipulating the chain conformations and properties of the polymer-modified interfaces since the delay in the brush response to an alternating voltage is only due to the relaxation time of the brush-forming chains.

A number of experimental studies [23,24,25,26,27] have demonstrated a pronounced response of polyelectrolyte brushes to applied voltage (i.e., an externally controlled electrostatic potential difference between the grafting surface and the bulk of the solution), though the interpretation of the results was far from being straightforward. Theoretical and computational studies [28,29,30,31,32] addressed primarily the conformations of strong (“quenched”) polyelectrolyte brushes grafted to charged surfaces.

A plausible hypothesis is that brushes formed by weak pH-sensitive polyelectrolytes could demonstrate a more pronounced responsiveness to electrical voltage rather than brushes of strong polyelectrolytes. The aim of the present study is to systematically compare the conformational responses of strong and weak polyelectrolyte brushes to applied electrical voltage.

The rest of the paper is organized as follows: After introducing the model of a planar polyelectrolyte brush, we start with a brief review of the electrostatic potential distribution and structural properties of brushes formed by strong and weak polyelectrolytes on neutral surfaces obtained analytically within the Poisson-Boltzmann strong-stretching (SS) approximation (Section 3). The results of the Scheutjens–Fleer self-consistent field (SF-SCF) numerical theory for strong and weak polyelectrolyte brushes exposed to applied voltage are presented in Section 4. Our conclusions are summarized in Section 5.

## 2. Model

**Figure 1:**
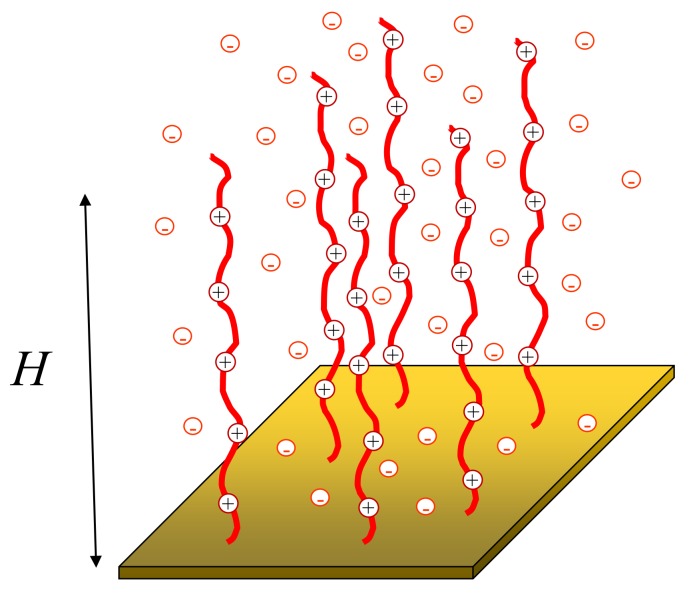
Schematics of a polyelectrolyte brush. *N* is the number of monomer units per chain, α-fraction of (positively) charged monomer uits, *s*-surface area per chain, *H*-brush thickness. Counterions localized predominantly inside the brush are depicted.

We consider a brush formed by polyelectrolyte chains with degree of polymerization (i.e., number of monomer uits per chain) *N*, Figure 1. The grafting density is $a^{2}/s$ where *s* is the area per chain on the grafting surface. The polyelectrolyte chains are assumed to be intrinsically flexible, that is, the monomer unit size *a* coincides with the statistical segment length of the corresponding uncharged polymer. The latter is assumed to be on the order of the Bjerrum length $l_{B}=e^{2}/\left(\epsilon k_{B}T\right)≅a$. The brush is in contact with a semi-infinite reservoir of aqueous solution which contains monovalent cations and anions with respective concentration $c_{+}=c_{-}=c_{s}$ that specifies the Debye screening length in the bulk of the solution as $\kappa^{-1}={\left(8\pi l_{B}c_{s}\right)}^{-1/2}$.

We consider separately brushes formed by strong (quenched) and weak (annealing or pH-sensitive) polyelectrolytes. Strong polyelectrolytes possess a quenched (positive) fractional charge α per monomer unit. In a weak polyelectrolyte brush every monomer units is capable to acqiure an elementary positive charge via protonation. The fraction α of ionized (protonated) monomer units in a weak polyelectrolyte brush depends on the pH in the bulk of the solution and the local electrostatic potential. The maximal value of $\alpha =\alpha_{b}$ that can be attained at a given solution pH is given by

(1) $$\alpha_{b}={\left(1+10^{pH-pK}\right)}^{-1}$$

where *K* is acidic ionization constant. A generalization of the theory for brushes made of negatively charged polyelectrolytes is straightforward.

## 3. Analytical Results for Polyelectrolyte Brushes Grafted to a Neutral Surface

We start with a brief review of the results of the analytical self-consistent field Poisson-Boltzmann theory developed earlier for brushes of strong (quenched) [15,16] and weak (annealing) [17] polyelectrolytes attached to a neutral planar surface and immersed into salt-containing solution. In this case the electrostatic potential difference between the grafting surface and the bulk of the solution (excess positive potential in the here-considered case of a polycationic brush) emerges due to electrical double layer formed by grafted polyions (immobilized charge) and non-uniformly distributed mobile counter- and coions of salt.

The theory implements the strong-stretching (quasi-classical) approximation to account for conformational entropy of the brush-forming chains introduced earlier by Semenov [33] combined with the Poisson-Boltzmann framework to describe Coulomb interactions between ionic species (charged monomer units and mobile co- and counterions). The strong stretching approximation assumes that the chains in the brush are extended with respect to their Gaussian dimensions and exhibit linear entropic elasticity on all length scales. As long as chain ends are distributed throughout the brush, which is the case for planar brushes of monodisperse linear chains in the absence of external fields, the self-consistent molecular potential acting on a monomer unit has the parabolic shape,

(2) $$\frac{U(x)}{k_{B}T}=-\lambda -\frac{3\pi^{2}}{8N^{2}a^{2}}x^{2},$$

where *x* is distance from the grafting surface and λ is a constant which depends on details of the interactions in the system. Since electrostatic interactions operating in the polyelectrolyte brush provoke strong stretching of the brush-forming chains, the SS approximation is sufficiently accurate except of the periphery of the brush formed by terminal non-stretched segments of the chains.

### 3.1. Strong Polyelectrolyte Brush, α = const

As long as Coulomb interactions dominate over short range excluded volume interactions in the brush, the self-consistent molecular potential given by Equation (2) is equal to the energy of the monomer unit in the self-consistent electrostatic potential $\Psi_{in}\left(x\right)$, i.e.,

(3) $$\frac{U(x)}{k_{B}T}=\frac{\alpha e\Psi_{in}\left(x\right)}{k_{B}T},$$

where subscript “in” refers to the interior of the brush, 0≤x≤H.

By combining Equations (2) and (3) the reduced self-consistent electrostatic potential, $\psi \left(x\right)\equiv e\Psi \left(x\right)/k_{B}T$, inside the brush (0≤x≤H) can be presented as [15,16]

(4) $$\psi_{in}\left(x\right)=\frac{H^{2}-x^{2}}{H_{0}^{2}}+C\left(\kappa \right),$$

where *x* is the distance from the grafting surface, *H* is the total thickness of the brush, $H_{0}$ is the characteristic length

(5) $$H_{0}=\sqrt{\frac{8}{3\pi^{2}}}N\alpha^{1/2}a$$

and constant C(κ) depends on the calibration of the potential and will be specified below.

By applying the Poisson equation,

(6) $$\frac{d^{2}\psi_{in}\left(x\right)}{dx^{2}}=-4\pi l_{B}\rho \left(x\right)$$

we can find the net charge density ρ(x) in the brush,

(7) $$\rho \left(x\right)={\left(2\pi l_{B}H_{0}^{2}\right)}^{-1}$$

and the residual (positive) charge per unit area

(8) $$\tilde{Q}=\int_{0}^{H}\rho \left(x\right)dx=\frac{H}{2\pi l_{B}H_{0}^{2}}.$$

The later is related to the Gouy-Chapman length

$$\tilde{\Lambda }=\frac{1}{2\pi l_{B}\tilde{Q}}=\frac{H_{0}^{2}}{H},$$

which controls the distribution of electrostatic potential and small mobile ions outside the brush, i.e., in the range x≥H.

Outside of the brush, at x≥H, the electrostatic potential $\psi_{out}\left(x\right)$ coincides with that for a uniformly charged plane with (positive) surface charge number density $\tilde{Q}$. It can be presented as [16]

(9) $$\psi_{out}\left(x\right)=2\ln\left[\frac{\left(\kappa \tilde{\Lambda }+\sqrt{{\left(\kappa \tilde{\Lambda }\right)}^{2}+1}-1\right)+\left(\kappa \tilde{\Lambda }-\sqrt{{\left(\kappa \tilde{\Lambda }\right)}^{2}+1}+1\right)e^{-\kappa (x-H)}}{\left(\kappa \tilde{\Lambda }+\sqrt{{\left(\kappa \tilde{\Lambda }\right)}^{2}+1}-1\right)-\left(\kappa \tilde{\Lambda }-\sqrt{{\left(\kappa \tilde{\Lambda }\right)}^{2}+1}+1\right)e^{-\kappa (x-H)}}\right].$$

The potential defined by Equation (9) vanishes at x→∞ whereas at the brush boundary, x=H, it has the value

(10) $$\psi_{out}\left(H\right)=-2\ln\left[\frac{\sqrt{{\left(\kappa \tilde{\Lambda }\right)}^{2}+1}-1}{\kappa \tilde{\Lambda }}\right].$$

The condition of continuity of the electrostatic potential at the edge of the brush, $\psi_{out}\left(H\right)=\psi_{in}\left(H\right)$, allows us to find constant C(κ) in Equation (4) as

(11) $$C\left(\kappa \right)=-2\ln\left[\frac{\sqrt{{\left(\kappa \tilde{\Lambda }\right)}^{2}+1}-1}{\kappa \tilde{\Lambda }}\right].$$

As one can see from Equations (4) and (11), the difference in the electrostatic potential between the grafting surface (x=0) and x=∞, ψ(0)−ψ(∞)=ψ(0), is equal to

(12) $$\psi \left(0\right)=\Delta \psi_{ref}=\frac{H^{2}}{H_{0}^{2}}+C\left(\kappa \right)=\frac{H^{2}}{H_{0}^{2}}-2\ln\left[\frac{\sqrt{{\left(\kappa \tilde{\Lambda }\right)}^{2}+1}-1}{\kappa \tilde{\Lambda }}\right]$$

and subscript “ref” here and below refers to the reference case of non-charged grafting surface. Notably the potential difference given by Equation (12) is due to the presence of polyelectrolyte chains end-grafted to the interface.

In the limit of low salt concentration in the solution, $\kappa \tilde{\Lambda }\ll 1$, we find $C\left(\kappa \right)\approx -2\ln\left(\kappa \tilde{\Lambda }/2\right)$, and the potential at the grafting surface ψ(0) increases with a decrease in salt concentration ($\kappa ∼c_{s}^{1/2}$) as

(13) $$\psi \left(0\right)=\Delta \psi_{ref}\approx \frac{H^{2}}{H_{0}^{2}}-2\ln\left(\frac{\kappa \tilde{\Lambda }}{2}\right)=\frac{H^{2}}{H_{0}^{2}}-2\ln\frac{\kappa }{2}-2\ln\left(\frac{H_{0}^{2}}{H}\right).$$

As follows from Equations (12) and (13), the potential diference arising due to the polyelectrolyte brush, $\Delta \psi_{ref}=\psi \left(0\right)$ is weakly affected by the degree of polymerization *N* of grafted polyions. This very weak increase in ψ(0) upon an increase in *N* is explained by accumulation of a larger amount of counterions in the brush with longer chains, and the corresponding reduction in the number of released counterions.

The difference of reduced electrostatic potential across the brush is given by

(14) $$\psi \left(0\right)-\psi \left(H\right)=\frac{H^{2}}{H_{0}^{2}},$$

and its typical value is on the order of unity which corresponds to ∼$10^{-1}$ V in absolute units.

Equations (12) and (14) are applicable in both salt-containing and salt-free solutions.

In the limit of low salt concentration (salt-free solution) $\kappa ∼c_{s}^{1/2}\to 0$, the concentration $c_{-}\left(x\right)$ of mobile counterions outside of the brush (i.e., at x≥H ) is given by

$$c_{-}\left(x\right)=\frac{1}{2\pi l_{B}{\left(x-H+\tilde{\Lambda }\right)}^{2}},x\geq H$$

and

(15) $$c_{-}\left(H\right)=\frac{1}{2\pi l_{B}{\tilde{\Lambda }}^{2}}=2\pi l_{B}{\tilde{Q}}^{2}={\left(2\pi l_{B}H_{0}^{2}\right)}^{-1}{\left(\frac{H}{H_{0}}\right)}^{2}.$$

Since the counterions are distributed according to the Boltzmann law, their concentration inside the brush is given by

(16) $$c_{-}\left(x\right)=c_{-}\left(H\right)\exp\left[\psi_{in}\left(x\right)-\psi_{in}\left(H\right)\right]={\left(2\pi l_{B}H_{0}^{2}\right)}^{-1}{\left(\frac{H}{H_{0}}\right)}^{2}\exp\left(\frac{H^{2}-x^{2}}{H_{0}^{2}}\right),$$

with the potential $\psi_{in}\left(x\right)$ defined by Equation (4). The polymer concentration profile $c\left(x\right)=[\rho \left(x\right)+c_{-}\left(x\right)-c_{+}\left(x\right)]/\alpha$ can be found by using Equation (7). The polymer concentration profile, c(x), and the distribution of chain free ends, g(x), can be conveniently presented using the reduced variables, $h=H/H_{0}$ and $t\left(x\right)=x/H_{0}$.

The polymer concentration profile is described by a truncated and shifted Gaussian function,

(17) $$\alpha c\left(x\right)={\left(2\pi l_{B}H_{0}^{2}\right)}^{-1}\left[1+h^{2}\exp\left(h^{2}-t^{2}\right)\right].$$

The distribution of the free ends is given by

(18) $$g\left(x\right)=\zeta^{-1}\frac{t}{H_{0}}\left[\frac{1+h^{2}}{\sqrt{h^{2}-t^{2}}}+\sqrt{\pi }\exp\left(h^{2}-t^{2}\right)\mathrm{erf}\left(\sqrt{h^{2}-t^{2}}\right)\right].$$

Here,

$$\mathrm{erf}\left(y\right)=\frac{2}{\sqrt{\pi }}\int_{0}^{y}\exp\left(-z^{2}\right)dz$$

is the error function, and parameter ζ is defined as

$$\zeta =H_{0}/\Lambda =\sqrt{\frac{3}{2}}\alpha^{3/2}l_{B}N^{2}a/s$$

A characteristic feature of the free end distribution given by Equation (18) is its divergency at the edge of the brush, at x=H. At x≪H it grows linearly with *x* and exhibits a single wide maximum in the central region of the brush.

The condition of conservation of the number of monomer units in the chain

$$s\int_{0}^{H}c\left(x\right)dx=N,$$

with the concentration profile c(x) given by Equation (17), allows for a closed equation for the reduced thickness of the brush, $h=H/H_{0}$,

(19) $$\zeta =h+h^{2}\frac{\sqrt{\pi }}{2}\exp\left(h^{2}\right),\mathrm{erf}\left(h\right)$$

which grows approximately linearly upon an increase in the degree of polymerization of the polyions *N* and is an increasing function of the fractional charge per monomer unit α and grafting density $a^{2}/s$.

In the case of salt-containing solution an analogous set of arguments leads to the following equation for the reduced brush thickness [16]

$$\zeta =h+\frac{\sqrt{\pi }}{8}{\left(\sqrt{{\left(\kappa H_{0}\right)}^{2}+h^{2}}+h\right)}^{2}\exp\left(h^{2}\right)\mathrm{erf}\left(h\right)$$

(20) $$+\frac{i\sqrt{\pi }}{8}{\left(\sqrt{{\left(\kappa H_{0}\right)}^{2}+h^{2}}-h\right)}^{2}\exp\left(-h^{2}\right)\mathrm{erf}\left(ih\right),$$

with $i^{2}=-1$, from which it follows that the brush thickness decreases as a function of salt concentration.

### 3.2. Weak (pH-Sensitive) Polyelectrolyte Brush

As demonstrated in ref [17], in the case of a weak (annealing) polyelectrolyte brush the self-consistent potential is related to the local degree of ionization α(x) by the equation

(21) $$\frac{U(x)}{k_{B}T}=\ln\left(1-\alpha \left(x\right)\right)$$

By combining Equations (2) and (21), we find an explicit expression for the profile of the degree of ionization in the brush as

(22) $$\alpha \left(x\right)=1-\left(1-\alpha_{H}\right)\exp\left[\frac{\alpha_{b}\left(H^{2}-x^{2}\right)}{H_{0b}^{2}}\right],$$

where

(23) $$H_{0b}=\sqrt{\frac{8}{3\pi^{2}}}N\alpha_{b}^{1/2}a,$$

and $\alpha_{H}\leq \alpha_{b}$ is the degree of ionization at the edge of the brush, i.e., at x=H. $\alpha_{H}$ depends in a complex way on the parameters of the system, i.e., $\alpha_{b}$ (controlled by pH, see Equation (1)), salt concentration $c_{s}$, chain length *N* and grafting density $a^{2}/s$.

Equations (21) and (22) apply to both weak polyacid and weak polybase brushes and as follows from Equation (22), the degree of ionization α(x) inside the brush monotonously increases as a function of *x*, i.e., with increasing distance from the grafting surface.

Because $\alpha_{b}$ is the degree of ionization of an individual monomer unit in the solution at infinite distance from the brush, where the electrostatic potential is zero, the mass action law allows to express the local degree of ionizaton α(x) inside the (cationic) brush through the local electrostatic potential $\psi_{in}\left(x\right)$ as

(24) $$\frac{\alpha (x)}{1-\alpha (x)}\cdot \frac{1-\alpha_{b}}{\alpha_{b}}=\exp\left(-\psi_{in}\left(x\right)\right).$$

(Note that the minus sign in the exponent on the r.h.s. of Equation (24) applies to a weak polycationic brush).

By combining Equations (22) and (24) we obtain the explicit form of the electrostatic potential profile inside a weak polybase brush as

(25) $$\psi_{in}\left(x\right)=\frac{\alpha_{b}\left(H^{2}-x^{2}\right)}{H_{0b}^{2}}-\ln\left\{\frac{1-\alpha_{b}}{\alpha_{b}\left(1-\alpha_{H}\right)}\left(1-\left(1-\alpha_{H}\right)\exp\left[\frac{\alpha_{b}\left(H^{2}-x^{2}\right)}{H_{0b}^{2}}\right]\right)\right\}.$$

Comparison of Equations (4) and (25) shows that the decay of the electrostatic potential $\psi_{in}\left(x\right)$ inside a weak polybase brush as a function of distance *x* from the grafting surface follows a more complex functional dependence on *x*, than in a strong (quenched) polyelectrolyte brush. At the edge of the brush the potential has the value

(26) $$\psi_{in}\left(H\right)=-\ln\frac{\alpha_{H}\left(1-\alpha_{b}\right)}{\alpha_{b}\left(1-\alpha_{H}\right)},$$

whereas its derivative allows to find the residual charge inside the brush as

(27) $$\tilde{Q}\left(H\right)=-\frac{1}{4\pi l_{B}}{\left(\frac{d\psi_{in}\left(x\right)}{dx}\right)}_{x=H}=\frac{1}{2\pi l_{B}}\frac{\alpha_{b}H}{\alpha_{H}H_{0b}^{2}}.$$

The excess potential created by the weak polycationic brush at the grafting surface with respect to the bulk of the solution is given by

(28) $$\psi_{in}\left(0\right)=\Delta \psi_{ref}=\frac{\alpha_{b}H^{2}}{H_{0b}^{2}}-\ln\left\{\frac{1-\alpha_{b}}{\alpha_{b}\left(1-\alpha_{H}\right)}\left(1-\left(1-\alpha_{H}\right)\exp\left[\frac{\alpha_{b}H^{2}}{H_{0b}^{2}}\right]\right)\right\},$$

and the potential difference across the brush is

(29) $$\psi \left(0\right)-\psi \left(H\right)=\frac{\alpha_{b}H^{2}}{H_{0b}^{2}}-\ln\left\{1-\left(1-\alpha_{H}\right)\exp\left[\frac{\alpha_{b}H^{2}}{H_{0b}^{2}}\right]\right\}$$

Finally, by equating electrostatic potentials $\psi_{in}\left(H\right)$ given by Equation (26) and $\psi_{out}\left(H\right)$ given by Equation (10) at the brush edge, x=H, we obtain a close equation for $\alpha_{H}$ in the form

$$\frac{\alpha_{H}\left(1-\alpha_{b}\right)}{\alpha_{b}\left(1-\alpha_{H}\right)}={\left(\frac{\sqrt{{\left(\kappa \tilde{\Lambda }\right)}^{2}+1}-1}{\kappa \tilde{\Lambda }}\right)}^{2},$$

where the Gouy–Chapman length outside the brush is given by

$$\tilde{\Lambda }=\frac{1}{2\pi l_{B}\tilde{Q}\left(H\right)}=\frac{\alpha_{H}H_{0b}^{2}}{\alpha_{b}H}.$$

## 4. Polyelectrolyte Brush Under Applied Electrical Field: Numerical SF-SCF Results

As discussed in the previous section, the presence of a polyelectrolyte brush leads to the appearance of excess (positive in the case of a polycationic brush) Coulomb potential at the grafting surface which itself is electroneutral. This excess voltage (relative to a zero reference voltage in the bulk of the solution) is on the order of $10^{-1}$ V and weakly depends on the length of grafted polyions. Connecting the surface to an external DC power source (with a reference electrode immersed in the solution) allows for tuning the electrical potential at the surface, i.e., the potential difference between the surface and the bulk of the solution. The shift of the surface potential with respect to the value created by the brush implies the appearance of positive or negative charge on the (conducting) substrate. This leads to conformational changes in the brush and, in the case of weak polyelectrolytes, also to a change in the ionization state of the polyelectrolyte chains.

Hence, if the potential difference between the grafting surface and the bulk of the solution (DC voltage) is used as external control parameter, at any potential difference larger or smaller than that given by Equations (13) and (29), an extra positive or negative charge density will be induced on the surface. It is important to note, that in the case of a polycationic brush, the surface potential remains positive even when the surface is weakly negatively charged. Only at a sufficiently large absolute value of immobilized negative charge the surface potential will change its sign.

In our numerical calculatios we use the immobilized surface charge density σ (measured in fractional number of elementary charges per unit area) as a control parameter, and calculate the potential difference Δψ(0) between the grafting surface and the bulk of the solution. This allows us to analyze the response of the polyelectrolyte brush to the applied external voltage which is used as a control parameter in experiments. Evidently, for a given σ the potential difference Δψ(0) depends on the polyelectrolyte brush parameters (N,α,1/s) and on the ionic stength (salt concentration $c_{s}$) and (in the case of weak polyelectrolyte brush) on the pH of the solution.

Below we use the reduced surface charge density defined as

(30) $$\gamma =\frac{\sigma }{\alpha N},$$

which is zero for a non-charged surface and acquires positive and negative values for positively and negatively charged surfaces, respectively. For a strong polyelectrolyte brush γ=−1 corresponds to the surface charge which exactly matches the bare charge of the brush-forming chains. For a weak polyelectrolyte brush, the actual degree of ionization depends on the surface charge and therefore in the following the value of α in Equation (30) is taken as the average fraction of ionized monomer units in the brush grafted to a neutral surface.

As discussed in our previous publications [28,29], the conformation of polyelectrolyte brushes grafted to a similarly or oppositely charged surface cannot be described within the analytical SS-SCF-framework based on parabolic molecular potential, (Equation (2)): The immobilized surface charge of the same sign creates an additional repulsive (stretching) force applied to the polyions leading to formation of a “dead zone” near the surface that is depleted of the chain ends. Moreover, the other hand, an oppositely charged grafting surface leads to partial adsorption of the chains from the brush and the strong stretching condition is violated for this population of polyions.

To analyze the conformational responses of strong and weak polyelectrolyte brushes to applied voltage we implement the numerical SF-SCF method which is free of the above mentioned limitations. The SF-SCF approach enables generating single-chain partition function of ionically charged poymer chain tethered onto the surface by iterative numerical solution of the Edwards diffusion equation. The composition law is used for calculating density distribution of monomer units which in turn gives rise to self-consistent molecular potential comprising contributions due to non-ionic (excluded volume) and ionic interactions. The short-range interactions are accounted for within Bragg-Williams approximation coupled to incompressibility condition. The Coulomb interactions betweenn all ionic species are treated within Poisson-Boltzmann approximation. A lattice implementation of the iterative scheme enables highly efficient and precise evaluation of structural and thermodynamic parameters of the system. The lattice site size is equal to the monomer unit length. In the case of polyelectrolyte brush grafted onto planar surface a one-gradient version of the SF-SCF algorithm assuming gradients of partial densities and fields only in the direction perpendicular to the grafting surface is emploied. More details about SF-SCF method and its application to planar polyelectrolyte brushes grafted onto charged surfaces can be found in ref [34] and in our original papers [28,29].

### 4.1. Strong Polyelectrolyte Brush, α = const

**Figure 2:**
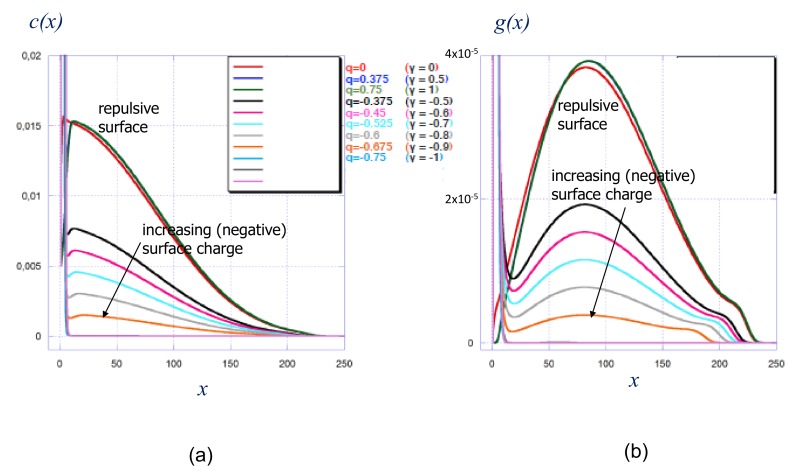
Strong polyelectrolyte brush: distribution of polymer density (**a**) and free chain ends (**b**) at various values of reduced surface charge density γ=σ/αN. Other parameters: N=300, α=0.5, $a^{2}/s=0.001$, salt concentration $c_{s}a^{3}=10^{-5}$.

In Figure 2a,b we present polymer density profiles and corresponding distributions of the chain ends in a strong polycationic brush in a salt-free solution at different values of reduced surface charge density γ.

At γ=0 corresponding to a non-charged surface, both polymer density profiles and free chain end distributions are in qualitative agreement with the predictions of the analytical theory, Equations (17) and (18): The polymer density profile, Figure 2a, exhibits the characteristic Gaussian shape, but, in contrast to Equation (17) decreases gradually at the edge of the brush. The peripheral decay in the polymer density is caused by the thermal fluctuations of the terminal chain segments that are not accounted for in the analytical SS-SCF theory. The chain end distribution, Figure 2b, grows approximately linearly with *x* close to the grafting surface and exhibits a broad maximum in the central region of the brush, in accordance with Equation (18). However, divergence in the end-point distribution at x=H predicted by Equation (18) and formally related to the jump-like drop in the polymer density (Equation (17)) is manifested in Figure 2b only as a pronounced shoulder close to the brush edge. This is again a consequence of fluctuations of the chain terminal segments that are not described by analytical SS-SCF theory but are properly accounted for by the SF-SCF method. We note that the shoulder in the chain end distribution is characteristic for polyelectrolyte brushes at low salt concentration.

At γ≥0 corresponding to positive surface charge density, σ≥0, and values of the surface potential $\Delta \psi \geq \Delta \psi_{ref}$, the structure of the brush is only weakly perturbed as compared to that of the brush grafted to the non-charged surface: Both the polymer density profile and chain end distribution extend slightly further away from the surface corresponding to the incremental increase in the stretching of the brush-forming chains, while a narrow but pronounced dead zone depleted from the chain ends appear close to the grafting surface. A further increase in γ does not lead to noticeable changes in the brush confirmation due to the screening of the positive surface by the cloud of counterions (anions) which is much thinner than the brush.

A qualitatively different conformational response of the brush is observed if the grafting surface is negatively charged, γ≤0. Recall that this regime corresponds to either positive, $0\leq \Delta \psi \leq \Delta \psi_{ref}$, or negative, Δψ≤0 surface potential. The polymer density profiles demonstrate enrichment of surface-proximal region with monomer units, due to electrostatically-driven adsorption of polycationic chains onto the negatively charged surface. Inspection of the chain end distribution, Figure 2b, confirms that at γ≤0 the tethered polyions segregate into two populations: adsorbed at the oppositely charged surface and stretched constituting the depleted brush with an effectively reduced number of the brush-forming chains per unit area (i.e., reduced effective grafting density). The intra-brush segregation was predicted in ref [29] on the basis of the analytical SS-SCF theory for polyelectrolyte brushes grafted onto the surface covered with an oppositely charge sub-layer of finite thickness that is penetrable for the polyelectrolyte chains and small ions. This prediction remains valid even in the limit of vanishing sublayer thickness (i.e., when SS-SCF approximation was not applicable).

**Figure 3:**
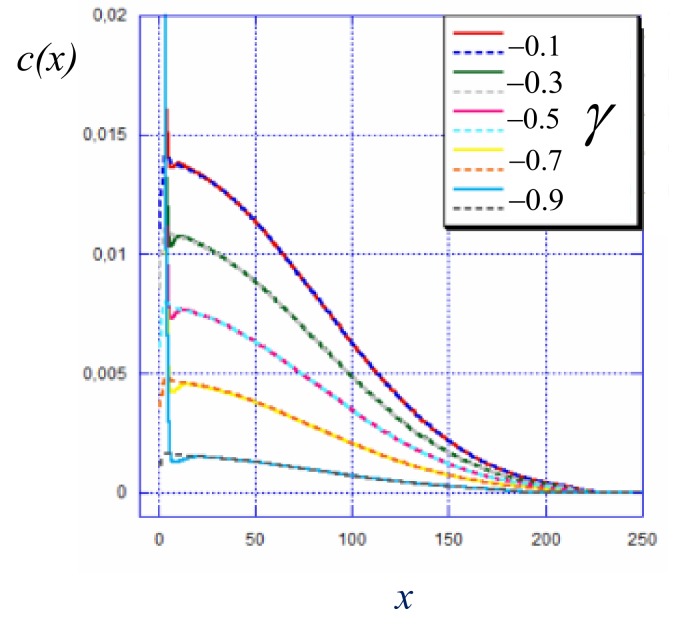
Polymer density profiles in a strong polyelectrolyte brush grafted onto an oppositely charged surface at varied reduced surface charge γ≤0 (as indicated), solid curves, and density profiles in depleted brushes with grafting density $1/s^{\prime }=\left(1-|\gamma |\right)/s$, dashed lines. Other parameters: N=300, $\alpha =0.5,a^{2}/s=0.001$, salt concentration $c_{s}a^{3}=10^{-5}$.

Moreover, it was demonstrated by the SS-SCF theory that the cumulative charge of the adsorbed polyelectrolyte chains matches the opposite charge immobilized on the surface. If this is the case, then the depleted brush formed by non-adsorped chains should have the same structure as a brush with reduced grafting density, $1/s^{\prime }=\left(1-|\gamma |\right)/s$, grafted to a non-charged surface. This conjecture is validated in Figure 3 in which the polymer density profiles in the brush grafted to the charged surface match in the central and distal regions of the density profiles of depleted brushes grafted to the non-charged surface. As one can see from Figure 2b, an increase in the absolute value of γ leads to progressive re-partitioning of the polyions from the population of stretched chains This process manifests itself in a progressive decrease of the area under the main maximum in the chain end distribution, and a decrease in the overall brush thickness. Notably, the end-point distribution maintains the characteristic shoulder in the peripheral zone of the brush.

**Figure 4:**
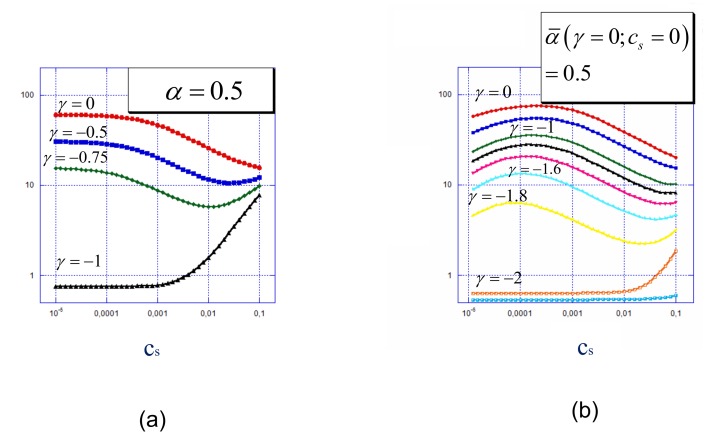
The average thickness, 〈H〉, (the first moment of the polymer density distribution) of strong (**a**) and weak (**b**) polyelectrolyte brushes as a function of salt concentration at different values of the surface charge density (as indicated at the curves). Other parameters: N=300, $a^{2}/s=0.001$.

The response of the brush to variations in the ionic strength of the solution depends on the sign and absolute value of the surface charge and is presented in Figure 4a. Here the average brush thickness, 〈H〉, measured as the first moment of the polymer density distribution is plotted as a function of salt concentration in the solution.

At positive or weakly negative γ, the brush structure in salt-free solution and its response to increasing salt concentration is qualitatively the same as for the brush tethered to a non-charged surface: The brush remains virtually intact as long as the concentration of added salt is smaller than the concentration of counterions entrapped inside the brush whereas at sufficiently high salt concentration the brush thickness noticeably decreases and then levels off at the value controlled by non-electrostatic (excluded volume) interactions. An opposite trend is observed at γ≈−1: In the absence of salt, all the chains are adsorbed onto the oppositely charged surface and form a thin and dense adsorption layer. An increase in salt concentration leads to the screening of the Coulomb attraction between the surface and the polyelectrolyte chains. This screening becomes efficient when the salt-controlled Debye length becomes smaller than the adsorbed layer thickness leading to progressive desorption of the tethered macroions.

The most peculiar dependence of the average brush thickness on salt concentration is predicted in the intermediate range of negative surface charge, γ≈−0.5 to −0.75. Here the polyions segregate into two approximately equal populations of adsorbed and extended chains. These two populations respond in different ways to increasing salt concentration in the solution. At moderate salt concentration the chains forming the depleted (and thus relatively sparse) brush experience the screening of repulsive Coulomb interactions by added salt that leads to a decrease in the extension of this population, whereas the population of adsorbed chains remain unaffected. As a result, the average brush thickness decreases. However, at high salt concentration, the salt-induced desorption of polyelectrolyte chains from the surface Altogether, the brush thickness passes through a minimum as a function of the salt concentration.

### 4.2. Weak vs. Strong Polyelectrolyte Brush

**Figure 5:**
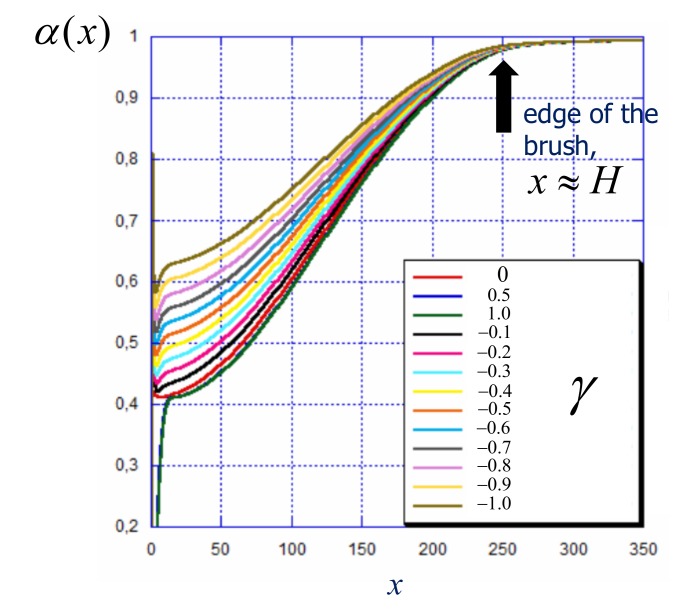
Profile of the degree of ionization in weak polyelectrolyte brushes as a function of distance *x* from the grafting surface at a varied value of the reduced surface charge γ. The pH−pK is set to adjust the average degree of ionization in the brush grafted to the neutral surface equal to 〈α〉=0.5. Other parameters: $N=300,a^{2}/s=0.001$, salt concentration $c_{s}a^{3}=10^{-5}$.

The specifics in the response of a weak polyelectrolyte brush to the applied voltage (or immobilized surface charge) emerge due to the shift in the ionization equilibrium compared to the reference brush grafted to a neutral surface. In Figure 5 we present the profiles of the local degree of ionization in a weak polyelectrolyte brush at different values of surface charge density. The pH−pK difference in the bulk of the solution is chosen in such a way, that in the reference state (non-charged surface) the average degree of ionization inside the brush is equal to 〈α〉=0.5. This value of 〈α〉 is also used in the calculation of the reduced surface charge γ according to Equation (30). As predicted by Equation (22) and can be seen in Figure 5, the degree of ionization is non-uniform inside the brush: It has the minimal value at the grafting surface and monotonously increases as a function of the distance from the grafting surface. Hence, due to excess (positive) local potential the ionization is strongly suppressed in the interior of the brush and approaches the value of $\alpha_{H}\leq \alpha_{b}$ close to the edge of the brush, x≈H. Therefore, at 〈α〉=0.5 the value of $\alpha_{b}$ is close to unity.

If the grafting surface is positively charged (γ≥0), the effect of the surface charge on the overall profile of the degree of ionization is minor, except for the immediate vicinity of the grafting surface. Here, the larger is the surface charge density, the stronger is the suppression of the brush ionization. As a result, the overall brush structure is also virtually unaffected by the positive charge of the surface. On the contrary, negative charge on the surface notably promotes ionization of the brush-forming chains primarily close the surface and also in the central region of the brush. As a result, the average degree of ionization and the overall bare charge of the brush increase.

**Figure 6:**
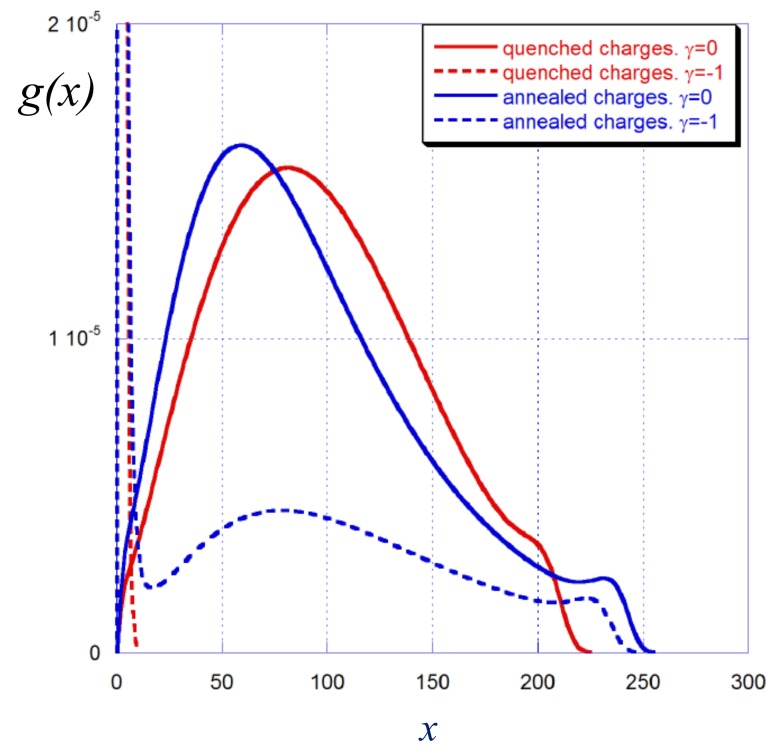
Distribution of the free chain ends in strong (red lines) and weak (blue lines) polyelectrolyte brushes grafted to a neutral surface (solid lines) and to the oppositely charged surface (dashed lines, γ=−1). The average fraction of charged monomer units in strong and weak polyelectrolyte brushes at zero surface charge is the same and corresponds to α=〈α〉=0.5. Other parameters: N=300, $a^{2}/s=0.001$, salt concentration $c_{s}a^{3}=10^{-5}$.

A fraction of the chains from the brush is adsorbed onto the oppositely charged surface and thus shields its charge. This is seen in Figure 6 in which the chain end distributions are presented for both strong and weak polyelectrolyte brushes grafted onto a neutral (solid curves) or an oppositely charged (dashed curves) surface. In the absence of charges on the surface the shape of the end-point distributions is qualitatively similar in strong and weak polyelectrolyte brushes, though in the latter case the shoulder in the peripheral region is more pronounced.

At γ=−1 the red dashed curves in Figure 6 that refer to a strong polyelectrolyte brush demonstrate the same trend as already discussed above: all the chains get adsorbed on the surface thus exactly matching its charge. In contrast, in a weak polyelectrolyte brush at γ=−1 two populations of polyelectrolyte chains are found: adsorbed (shielding the surface charge) and extended (forming a depleted brush with decreased thickness). Because at γ=−1 the surface charge exactly matches the charge of polyions grafted to the neutral surface, in the presence of negative surface charge the brush undergoes extra ionization and acquires additional positive charge so that the bare brush charge overcompensates the surface charge.

**Figure 7:**
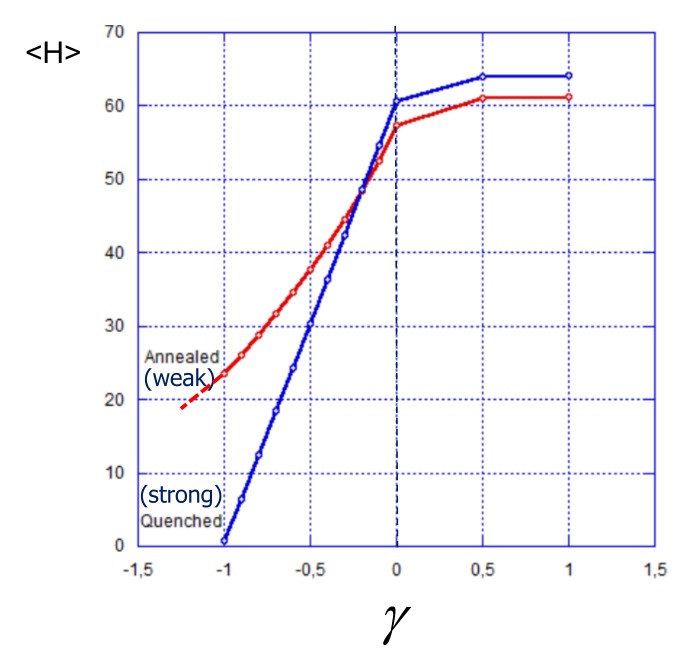
Average thickness (the first moment of the polymer density) in strong (blue curve) and weak (red curve) polyelectrolyte brushes as a function of reduced surface charge density γ. The average fraction of charged monomer units in strong and weak polyelectrolyte brushes at zero surface charge is the same and corresponds to α=〈α〉=0.5. Other parameters: $N=300,a^{2}/s=0.001$, salt concentration $c_{s}a^{3}=10^{-5}$.

The effect of enhanced ionization of a weak polyelectrolyte brush by an oppositely charged surface is also manifested in the dependence of the average brush thickness on the surface charge presented in Figure 7. As one can see in Figure 7, at γ≥0, i.e., at zero or positive charge on the surface, the average thickness of the strong polyelectrolyte brush is slightly larger than that of the weak one, and in both cases, the thickness only weakly increases as a function of the surface charge γ exhibiting saturation at γ≫1. As discussed above, shielding of the surface charge by a thin cloud of counterions makes the brush insensitive to the magnitude of the surface charge in this regime. A different behavior is observed in the range of negative surface charge: Here the thickness of the strong polyelectrolyte brush decreases more sharply as a function of γ approaching a value close to zero (corresponding to a thin adsorbed polyelectrolyte layer) at γ→−1. A further decrease in γ leads only to the compaction of the adsorbed layer. In contrast, the thickness of a weak polyelectrolyte brush decreases more gradually and remains much larger than the thickness of a strong polyelectrolyte brush, and comparable to the thickness of the brush grafted to a neutral surface even at γ≈−1. The thickness of the weak polyelectrolyte brush in this regime is controlled by a significant population of stretched chains (seen in Figure 6), whereas the surface charge is matched and shielded by a fraction of the brush polyions which are more strongly ionized than in a reference brush grafted to a neutral surface. Hence, even though strong and weak polyelectrolyte brushes have approximately equal thicknesses in the reference state (neutral substrate), the weak one remains much thicker than the strong one at the same value of immobilized negative surface charge.

A weak polyelectrolyte brush grafted to a charged interface demonstrates very rich behavior as a function of the solution salt concentration $c_{s}$, Figure 4b. The shape of the dependence of the brush thickness on salt concentration changes upon variation in the surface charge density. At zero or positive surface charge the 〈H〉 vs. $c_{s}$ dependence exhibits a characteristic non-monotonous shape predicted earlier for weak polyelectrolyte brushes on a neutral surface: an initial increase in the brush thickness at low salt concentration is provoked by enhancing ionization of the brush-forming chains and is followed by the decrease at high salt concentration when the brush ionization reaches the $\alpha_{b}$ level and a further increase in salt concentration leads to screening of intermolecular Coulomb repulsions.

The same shape of the 〈H〉 vs. $c_{s}$ dependence is preserved up to relatively large (in absolute value) negative surface charge density, though the thickness of the brush at any $c_{s}$ systematically decreases upon a decrease in γ due to an increasing fraction of adsorbed chains (moderated by a concomitant increase in their degree of ionization). At sufficiently negative γ the fraction of adsorbed chains becomes significant and the desorption of polyions provoked by the screening of Coulomb attraction to the surface at a high salt concentration leads to an increase in the brush thickness. Altogether, in a certain range of (negative) γ the 〈H〉 vs. $c_{s}$ dependence may exhibit both a maximum and a minimum upon a progressive increase in the salt concentration. Such complex dependency is not feasible for strong polyelectrolyte brushes.

### 4.3. Relationship Between Surface Charge and Surface Potential

In experiments, the surface potential rather than the surface charge density is implemented as a control parameter. Due to adjustable ionization of pH-sensitive polyions, strong and weak polyelectrolyte brushes exhibit different conformational responses to the same surface charge. Therefore, the same value of surface charge corresponds to different values of the electrostatic potential at the surfaces decorated by strong and weak polyelectrolyte brushes.

**Figure 8:**
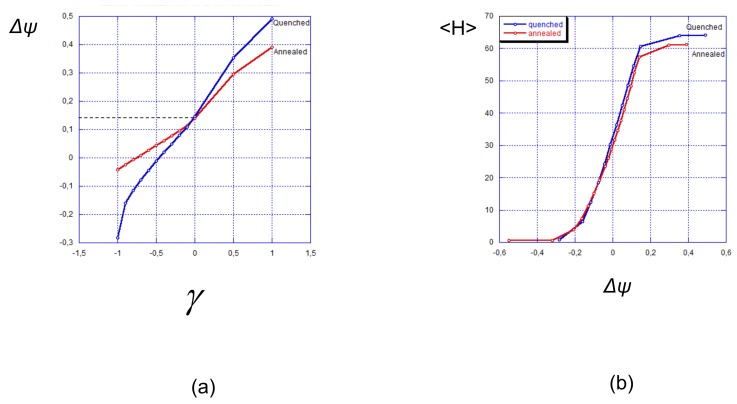
Surface potential Δψ vs. reduced surface charge γ (**a**) and average thickness (the first moment of the polymer density) vs. surface potential in strong (blue curves) and weak (red curves) polyelectrolyte brushes (**b**). The average fraction of charged monomer units in strong and weak polyelectrolyte brushes at zero surface charge is the same and corresponds to α=〈α〉=0.5. Other parameters: $N=300,a^{2}/s=0.001$, salt concentration $c_{s}a^{3}=10^{-5}$.

This is illustrated by Figure 8a, where the surface potential is presented as a function of the reduced surface charge density γ. As expected, at γ=0 (neutral surface) the surface potential has a positive value of approximately +0.15 V due to the presence of a polycationic brush. Notably, it is almost the same for strong and weak polyelectrolyte brushes. Positive or negative charges immobilized on the surface provoke the increase or decrease in the surface potential, respectively. In both positive and negative ranges of γ the surface potential varies more steeply in the case of a strong polyelectrolyte brush compared to the case of a weak one.

Compared to the reference state (neutral surface) a positively charged surface with γ≥0 suppresses the ionization of a weak polycationic brush and the overall positive charge of the interface is smaller than (σ+〈α〉N/s). On the contrary, a negatively charged surface promotes ionization (protonation) of the brush-forming chains and thus shifts the surface potential in a positive direction. The surface potential equals zero at a certain value of negative surface charge density. For a weak polyelectrolyte brush vanishing of the surface potential occurs at a larger absolute value of negative surface charge as compared to that for a strong one.

By using the data for the average brush thickness 〈H〉 and surface potential Δψ in Figure 7 and Figure 8a, we find the dependece of 〈H〉 on the surface potential Δψ, which is presented in Figure 8b vs. γ. As it is seen in Figure 8b the difference in the dependences of 〈H〉 on Δψ for strong and weak polyelectrolyte brushes is much less pronounced than one could have expected. This is a consequence of two effects: weaker dependence of 〈H〉 on γ for a weak polyelectrolyte brush (Figure 7) and stronger dependence of Δψ on γ (Figure 8a) for a weak polyelectrolyte brush compared to a strong one.

Altogether, a strong polyelectrolyte brush exhibits a slightly steeper dependence of 〈H〉 on Δψ than a weak one. The difference becomes more pronounced when the (average) degree of ionization in both brushes at γ=0 decreases.

## 5. Conclusions

In this paper, we used the numerical SF-SCF method to investigate the behavior of strong and weak polyelectrolyte brushes grafted to solid-liquid interface with either immobilized surface charge or applied surface potential. The conformational structure of the brush in the electrical field is compared to the reference state with zero surface charge, that can be described using the analytical SS-SCF method.

In both cases of strong and weak polycationic brushes the surface potential at zero surface charge (reference state) is on the order of $10^{-1}$ V, weakly depends on the length of grafted polyions and decreases upon an increase in salt concentration. This points to the characteristic magnitude of voltage from an external source that is necessary to apply for manipulating the brush conformation. The surface potential vanishes only when the surface is sufficiently strongly negatively charged. The same holds (with the inverse sign of the potential) for polyanionic brushes.

Both strong and weak polycationic brushes grafted onto a negatively charged surface exhibit segregation in two populations: (i) polyions adsorbed on the surface whose total charge matches the surface charge, and (ii) extended chains forming a depleted polyelectrolyte brush. Since the adsorbed population essentially neutralizes the surface charge on a length scale much smaller than the thickness of the depleted brush, the latter has a structure similar to that in a polyelectrolyte brush with smaller grafting density. This effect has been previously predicted [29] within the SS-SCF approach for strong polyelectrolyte brushes grafted to the surface covered with an oppositely charged sublayer (e.g., a ’surface-immobilized film of polyelectrolyte gel). We demonstrate here that intra-brush segregation takes place also for weak polyelectrolytes, but with significant modification: Because an oppositely charged surface provokes extra ionization of tethered macroions, the brush stratification is retained even if the surface charge density exceeds the bare charge of the brush (per unit area) in the reference state (i.e., at zero surface charge).

The thickness of a stratified weak polyelectrolyte brush can exhibit a peculiar dependence on the salt concentration: it may increase at low salt concentration, pass through a maximum, and decrease at intermediate salt concentration, pass through a minimum and then increase again at high salt concentration. The initial and final salt-induced increases in the brush thickness have different physical origins: The former is due to the promoted ionization of polyions in the extended population, whereas the latter is due to desorption of the chains from the adsorbed population.
